# Gastric inverted polyp successfully resected by underwater endoscopic mucosal resection

**DOI:** 10.1002/deo2.70103

**Published:** 2025-03-23

**Authors:** Yuki Nakamura, Takahiro Nomi, Sho Yasui, Jun‐ichi Okano, Ichiro Yamadori, Hidetaka Yamamoto, Yuichiro Sasaki, Hajime Isomoto

**Affiliations:** ^1^ Department of Gastroenterology Saiseikai Sakaiminato General Hospital Tottori Japan; ^2^ Department of Pathology Fukuyama Medical Association Health Support Center Hiroshima Japan; ^3^ Department of Pathology and Oncology Okayama University Graduate School of Medicine, Dentistry, and Pharmaceutical Sciences Okayama Japan; ^4^ Department of Multidisciplinary Internal Medicine Division of Medicine and Clinical Science Tottori University Faculty of Medicine Tottori Japan

**Keywords:** clinicopathologic characteristic, gastric inverted polyp, gastric subepithelial lesions, stromal proliferation, underwater endoscopic mucosal resection

## Abstract

A 47‐year‐old man, who underwent an annual esophagogastroduodenoscopy as part of his routine check‐up, was found to have a small subepithelial lesion on the posterior wall of the gastric body in 2013. The lesion gradually increased in size during follow‐up. In 2024, 11 years after the initial detection, the lesion had grown to over 10 mm in diameter and exhibited central erythematous depression. Endoscopic ultrasonography was performed, revealing that the lesion originated from the second layer with uniformly low internal echogenicity. A biopsy suggested the possibility of a benign stromal tumor, but a definitive diagnosis was not achieved. To obtain a complete biopsy, en bloc resection was performed using underwater endoscopic mucosal resection. Histopathological examination confirmed the diagnosis of a gastric inverted polyp. Since gastric inverted polyps with central depression or erosion have the potential for malignancy, endoscopic resection is recommended.

## INTRODUCTION

Gastric inverted polyps (GIPs) are rare gastric polyps characterized by the submucosal inversion of mucosal components. They are most commonly referred to as “inverted hyperplastic polyps” or “hamartomatous inverted polyps” in the current literature. GIPs exhibit a wide range of morphologic variations. Kim et al^1^. classified GIPs into three subtypes based on their connection to the mucosal surface, the presence of a smooth muscle boundary, and their tissue organization patterns, and described differing morphological features and clinical behaviors for each subtype.

Underwater endoscopic mucosal resection (UEMR) is a novel technique that serves as an alternative to conventional endoscopic mucosal resection (EMR)^2^. Recent studies have reported UEMR as a safe and effective treatment for upper gastrointestinal subepithelial lesions (SELs) located within the submucosal layer^3^.

We present a case of a GIP that exhibited progressive growth over more than 10 years of follow‐up and was successfully resected using UEMR.

## CASE REPORT

**FIGURE 1:**
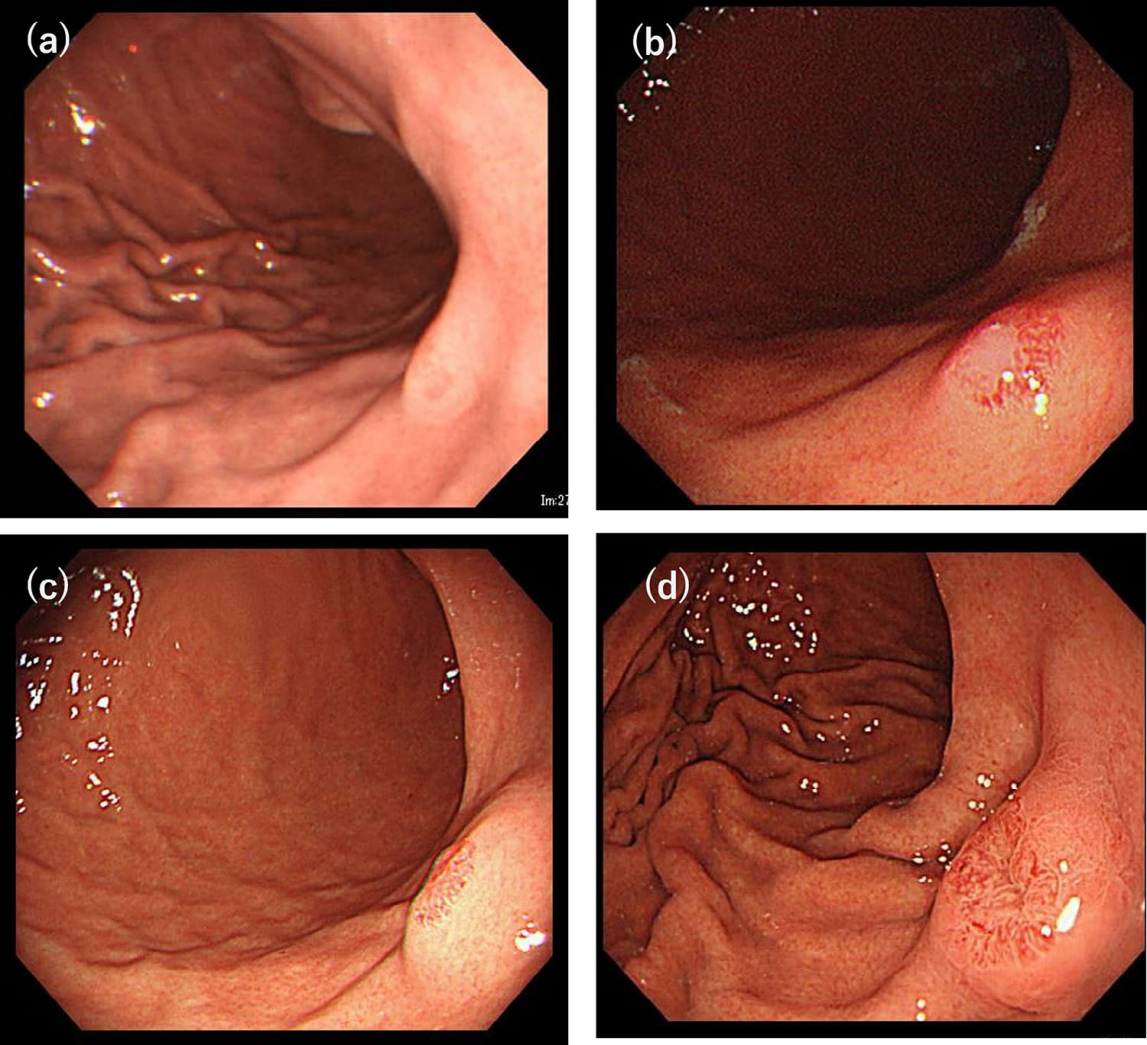
Series of esophagogastroduodenoscopy examinations showing progressive growth of the lesion over time. (a) 11 years prior. (b) 7 years prior. (c) 4 years prior. (d) At the time of resection, esophagogastroduodenoscopy revealed a gastric subepithelial lesion over 10 mm in diameter with central erythematous depression on the posterior wall of the gastric body

**FIGURE 2:**
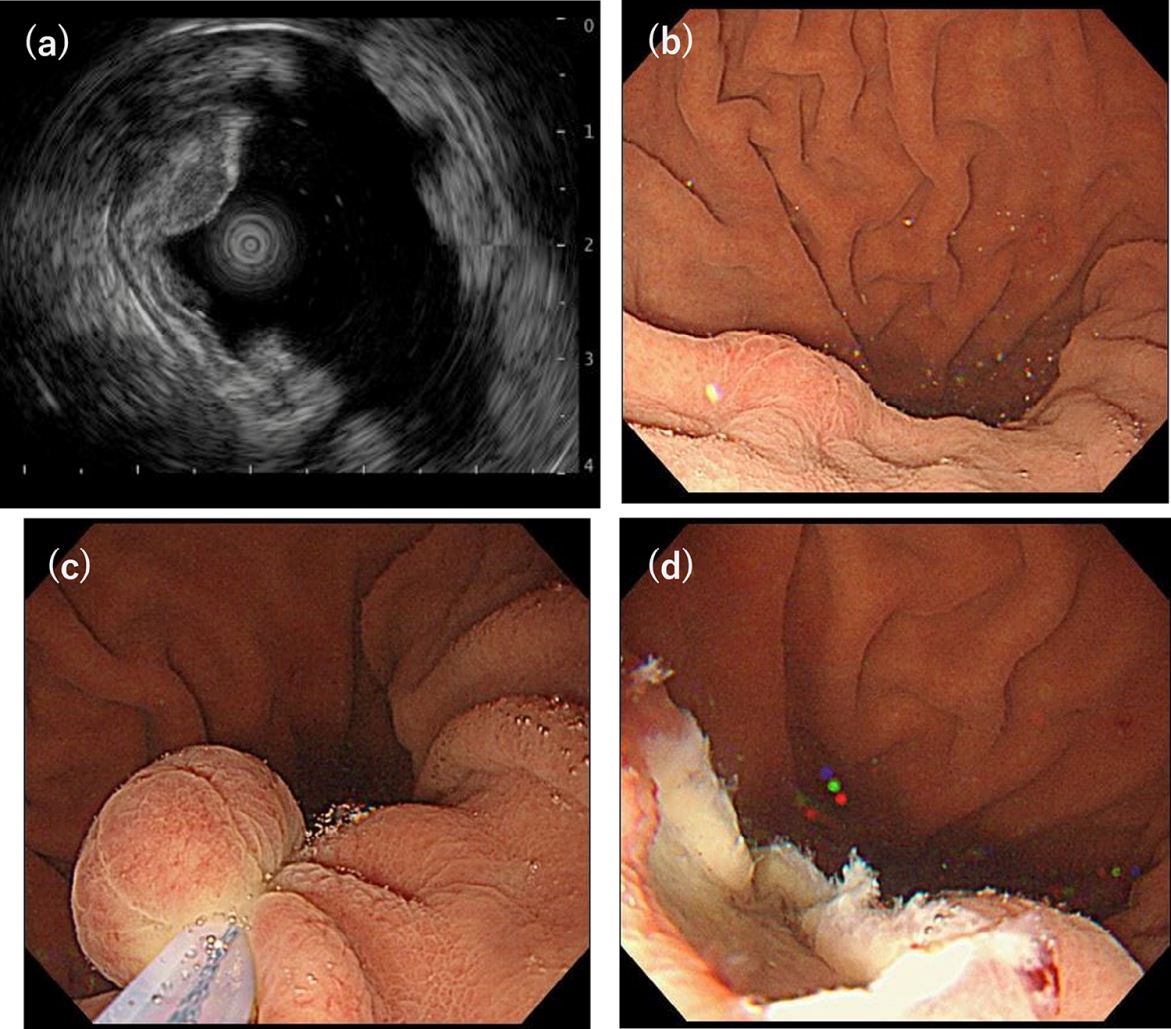
(a) Endoscopic ultrasonography demonstrated a low‐echoic mass originating from the second layer of the gastric wall, with no disruption of the third layer. (b–d) Water filling in the lumen caused the lesion to float, facilitating easy snaring of the tumor

A 47‐year‐old man had been undergoing routine esophagogastroduodenoscopy annually since 2011, and a SEL was first detected in 2013 (Figure 1a). The lesion measured approximately 5 mm or less in diameter, and we opted for annual endoscopic surveillance. During follow‐up, the lesion exhibited a gradual increase in size (Figure 1b,c). In the 2017 endoscopy (Figure 1b), a biopsy was taken from a slightly depressed area with erosion, revealing hyperplastic changes without malignancy. Based on its morphology, we suspected the lesion to be a gastric aberrant pancreas. In 2024, esophagogastroduodenoscopy revealed that the SEL had grown to over 10 mm in diameter, with central erythematous depression (Figure 1d). The gastric mucosa showed no atrophy, and the patient tested negative for serum anti‐*Helicobacter pylori* antibody. Endoscopic ultrasonography (EUS) demonstrated that the lesion appeared as a homogeneously low‐echoic mass originating from the second layer of the gastric wall, with no disruption of the third layer (Figure 2a). A biopsy of the erythematous and eroded surface suggested a benign mesenchymal tumor; however, a definitive diagnosis was not achievable.

**FIGURE 3:**
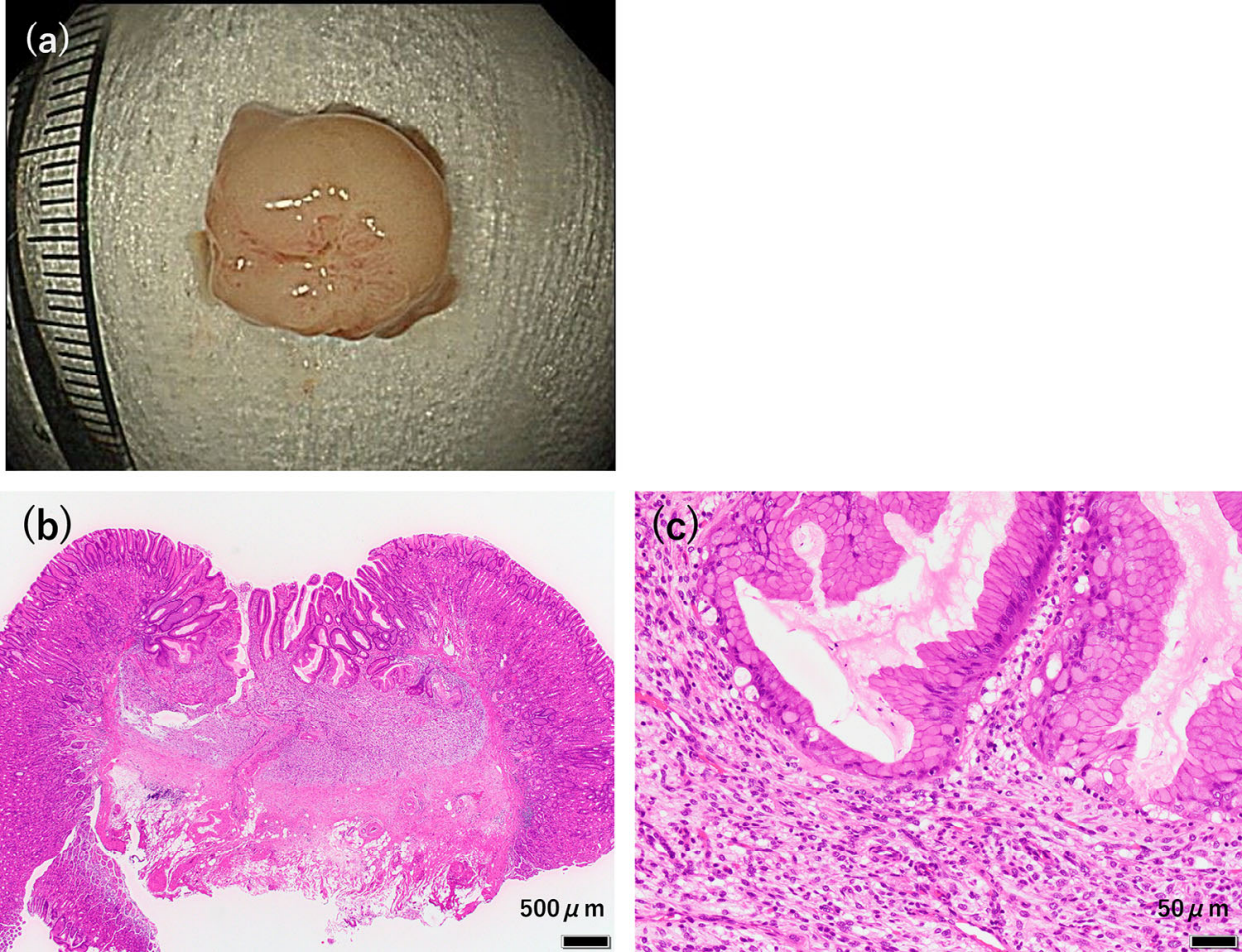
(a) En bloc resection was successfully achieved. (b, c) Histological analysis of resected specimen (10×8 mm). Hematoxylin and eosin staining: (b) Low‐power magnification showing hyperplastic glandular epithelium within the mucosa, inverted and proliferating into the submucosa (scale bar: 500 µm). (c) Medium‐power magnification showing spindle‐cell proliferation (scale bar: 50 µm)

**FIGURE 4:**
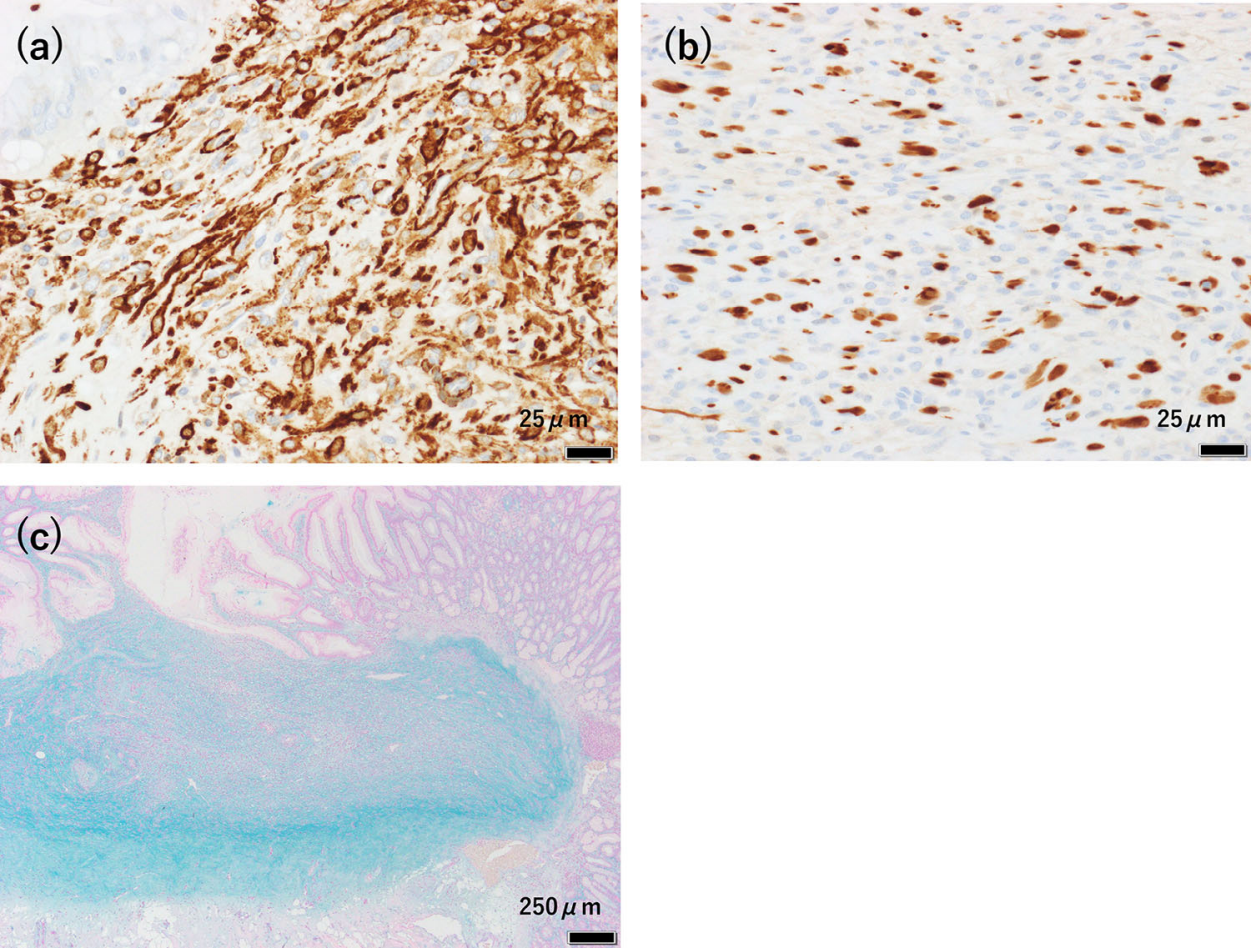
Immunohistochemical staining; (a) The spindle‐shaped cells were positive for α‐smooth muscle actin (scale bar: 25 µm). (b) The spindle‐shaped cells were partially positive for S‐100 (scale bar: 25 µm). Alcian blue staining: (c) The myxoid stroma was highlighted (scale bar: 250 µm)

Given the tumor's gradual increase in size over time, we considered the possibility of malignant potential. Based on the EUS findings, we deemed endoscopic resection feasible. UEMR was performed using an Endocut Q current (effect 3; cut duration 2; cut interval 3) generated by a VIO300S (ERBE Elektromedizin GmbH; Figure 2b–d). The lesion was successfully resected en bloc, with negative lateral and deep resection margins (Figure 3a). Histopathological analysis revealed that hyperplastic glandular epithelium within the mucosa had inverted and proliferated into the submucosa. Spindle‐shaped cells with minimal atypia proliferated within the lamina propria and submucosal layers, surrounded by fibromyxoid stroma (Figure 3b,c). Immunohistochemical staining showed that the spindle‐shaped cells were positive for α‐smooth muscle actin (Figure 4a) and partially positive for S‐100 (Figure 4b), while negative for c‐kit, CD34, desmin, HMB45, and cytokeratin AE1/AE3. The MIB‐1 index was low. Alcian blue staining highlighted the myxoid stroma (Figure 4c). Based on these findings, the lesion was diagnosed as a GIP with stromal proliferation.

## DISCUSSION

GIPs are a rare type of gastric polyp characterized by the submucosal inversion of mucosal components^1^. In this case, the initial biopsy specimen taken from the tumor surface suggested a benign stromal tumor. However, after excisional resection, histological examination revealed spindle cell proliferation with fibromyxoid stroma in the lamina propria and submucosal layers. Additionally, hyperplastic glandular epithelium was observed to have inverted and proliferated into the submucosa, which is a hallmark feature of GIPs. Immunohistochemical staining ruled out a specific submucosal tumor, confirming the diagnosis of a GIP with stromal proliferation. Diagnosing GIPs based solely on biopsy is challenging, as an accurate diagnosis requires full visualization of the lesion's architecture. In this case, the biopsy specimen was difficult to diagnose because it primarily highlighted stromal proliferation, which obscured the overall structural features of the lesion. Consequently, complete resection is essential for a definitive and accurate diagnosis.

Kim et al^1^. classified GIPs into three subtypes based on histopathological features: type 1 is characterized by a central mucosal communication and a well‐defined smooth muscle boundary, which can be endoscopically visualized as a central orifice or depression. Type 2, is similar to type 1 but lacks central communication. Type 3 lacks both a central communication and a smooth muscle boundary and features a lobular organization of cystic or hyperplastic glands with smooth muscle proliferation. Given that this case displayed continuity between the submucosal glands and the surface mucosa, it was classified as a type 1 GIP. In some previous reports, the central communicating structure in type 1 GIPs can be visualized as internal ducts or tubular structures on EUS,^4^. but these findings were not observed in this case.

In a review of 12 cases by Kim et al.,^1^ six were classified as type 1 GIPs, three of which demonstrated stromal proliferation, similar to the present case. Additionally, half of the type 1 GIPs were associated with adenocarcinoma, presenting as hyperemic elevation or erosion on endoscopy. Notably, all cases with coexisting carcinoma were smaller than 2.0 cm. In contrast, no coexisting carcinomas were identified in type 2 or type 3 GIPs. Since GIPs are characterized by the downward growth of hyperplastic mucosal components, their pathogenesis is presumed to be similar to that of hyperplastic polyps. In the present case, the absence of *H. pylori* infection and long‐term proton pump inhibitor use suggests that chronic inflammation from mechanical irritation due to peristalsis may have contributed to GIP development. Regarding the malignant transformation, the multistep carcinogenesis theory has been reported in hyperplastic polyps^5^. Kim et al^1^. also described hyperplastic‐dysplasia‐carcinoma sequences within cancerous type 1 GIPs, hypothesizing that carcinomatous transformation might occur due to the central mucosal communication, which could expose lesional epithelial cells to luminal carcinogens and mechanical stress.

In this case, the preoperative diagnosis was a mesenchymal tumor based on the biopsy results. EUS revealed that the lesion originated from the second layer of the gastric wall and measured approximately 15 mm in diameter without disruption of the third layer. Given these findings, endoscopic resection was deemed appropriate. Several endoscopic techniques are available for resection of SELs, with the method chosen depending on the lesion's size and layer of origin^6^. EMR using a ligation device has been reported as a method for resecting small SELs originating from the submucosa^7^. While simple, safe, and effective, this method is limited by the size of the lesion, as a larger lesion (>10 mm) cannot be fully resected within the ligation band. EMR may flatten or bury small lesions or those extending into the submucosa by submucosal saline solution injection, complicating snare capture. Endoscopic submucosal dissection, while suitable for en‐bloc resection with deep negative margins, requires more advanced technical skill and longer procedure time than EMR, which can lead to increased complications and higher medical costs^8^.

UEMR is a technique that uses a hot snare with electrocautery to remove gastrointestinal polyps without the need for submucosal injection while filling the lumen with water^2^. This method has demonstrated efficacy in the removal of rectal neuroendocrine tumors ≤10 mm, achieving R0 resection rates comparable to those of endoscopic submucosal dossection.^9^ Kim et al.^3^ also reported its efficacy and safety for upper gastrointestinal SELs. In their review of 17 submucosal lesions without muscularis propria invasion, they observed an average lesion size of 9 mm (range: 3–15 mm), an average procedure time of 3.2 min (range: 1.3–8.7 min), and a 100% en bloc and complete resection rate. Based on these findings, UEMR is considered suitable for SELs ≤15 mm without muscle layer invasion. In this case, the lesion was a relatively small SEL, although it exceeded 10 mm in diameter, UEMR resection was chosen for resection and complete resection was achieved without complications.

In conclusion, given this case and previous literature, endoscopic resection is recommended for small type 1 GIPs, as they have the potential for enlargement and malignant transformation. UEMR is a safe and effective method and may be a viable option for resecting GIPs.

## CONFLICT OF INTEREST STATEMENT

None
